# Methotrexate for the Treatment of Graft-versus-Host Disease after Allogeneic Hematopoietic Stem Cell Transplantation

**DOI:** 10.1155/2014/980301

**Published:** 2014-10-27

**Authors:** Amr Nassar, Ghada Elgohary, Tusneem Elhassan, Zubeir Nurgat, Said Y. Mohamed, Mahmoud Aljurf

**Affiliations:** ^1^National Research Center, Tahrir Street, Cairo, Egypt; ^2^Adult HSCT Program, Ain Shams University Hospitals, P.O. Box 1156, Cairo, Egypt; ^3^Adult HSCT Program, Oncology Center, King Faisal Specialist Hospital & Research Center, P.O. Box 3354, Riyadh 11211, Saudi Arabia; ^4^Pharmaceutical Care Division, King Faisal Specialist Hospital & Research Center, P.O. BOX 3354, Riyadh 11211, Saudi Arabia

## Abstract

Glucocorticoids have been the primary treatment of graft-versus-host disease (GVHD) over the past decade. Complete responses to steroid therapy are usually expected in almost one-third of aGVHD cases and partial response is anticipated in another one-third of patients. However, for those patients not responding to corticosteroid treatment, there is no standard second-line therapy for acute or chronic GVHD. Methotrexate (MTX) for treatment of steroid refractory GVHD has been evaluated in a number of studies. Results from peer-reviewed original articles were identified and the pooled data analyzed. Despite several limitations in data collection and analysis, weekly administration of methotrexate at a median dose of 7.5 mg/m^2^ seems to be safe with minimal toxicities in the context of both aGVHD and cGVHD treatments. The observed overall response (OR) in patients with aGVHD to MTX treatment in the published studies was 69.9%, with complete response (CR) in 59.2% and PR in 10.6%. In cGVHD the OR was 77.6%, with CR reported in 49.6% and PR in 28% of patients. Predictors of better responses were lower grade GVHD, cutaneous involvement, and isolated organ involvement. MTX as a steroid sparing agent might reduce long-term complications and improve the quality of life of GVHD affected individuals.

## 1. Introduction

Use of allogeneic stem cell transplant (allo-SCT) as a therapeutic option for otherwise lethal diseases is continuously increasing [1]. However, graft-versus-host disease (GVHD) remains a major complication of allo-SCT, affecting up to 40–60% of allo-SCT patients [2]. GVHD occurs when immune competent cells, namely, T-lymphocytes, recognize membrane antigens on the donor cells. These membrane antigens include a set of host polypeptides such as major and minor histocompatibility antigens displayed by the human leukocyte antigen system. The polymorphism of these polypeptides triggers T-cell activation and ultimately tissue injury through a variety of cellular effector mechanisms. The activation of the donor immune cells is augmented also by cytokines released from the site of tissue injury associated with the intense conditioning regimen (cytokine storm) [3].

Acute GVHD (aGVHD) usually occurs in the first 100 days after transplantation, whereas onset of chronic GVHD (cGVHD) is observed later. Changes in the onset period of both acute and chronic GVHDs have been observed, with acute cases occurring 100 days after transplantation and chronic cases noticed earlier than usual. These changes from traditional patterns of acute and chronic GVHD were observed especially in the context of reduced conditioning intensity and use of peripheral blood as a stem cell source [4–6].

Over the years, several methods for GVHD prophylaxis and treatment, such as immunosuppressive medications, graft engineering, and cellular therapies, have been explored [7–14]. Calcineurin inhibitors and methotrexate (MTX) combination therapy was used successfully to reduce the incidence and severity of GVHD and is the standard of care for GVHD prophylaxis [15, 16]. Successful GVHD prophylaxis was also reported using combinations with mycophenolate mofetil, sirolimus, and other immunomodulatory agents [17]. Other approaches used to reduce the risk of GVHD include use of nonmyeloablative conditioning and gut decontamination to reduce tissue damage and cytokine storm that might trigger GVHD and biological agents that can modify the cytokine and immune response after allo-SCT [18–24].

Glucocorticoids are the mainstay of aGVHD therapy due to their Lymphocytic and anti-inflammatory properties, and these compounds have been the primary treatment of GVHD for more than 3 decades [25].

Complete responses to steroid therapy are usually expected in almost one-third of aGVHD cases after human leukocyte antigen (HLA) matched sibling donor allo-SCT, and partial response is anticipated in another one-third of patients [25, 26]. However, a lower response rate is observed after unrelated donor allo-SCT [24]. Currently, there is no standard second-line therapy for acute or chronic GVHD, and several candidate drugs were tested. Supplementation of steroid therapy with agents such as antibodies against IL-2R, antithymocyte globulin (ATG), etanercept, and infliximab did not significantly improve survival over single agent steroids. Subsequently, corticosteroids remain the standard of care for initial treatment of aGVHD [27–30]. Steroid refractory acute and or chronic GVHD still represents a major therapeutic challenge [22]. Furthermore, development of steroid sparing agents to be used either in combination with steroids or in salvage therapy is a top priority in the treatment of acute or chronic GVHD due to the short- and long-term complications associated with steroids use [24].

MTX, one of the earliest drugs used for GVHD prophylaxis, inhibits dihydrofolate reductase and production of thymidylate and purines, thereby suppressing T-cell response and proliferation as well as expression of adhesion molecules [31–33]. Although MTX was widely applied for GVHD prophylaxis, only few published trials addressed its efficacy in the treatment of acute and chronic GVHD [34–41]. In this review, we pooled data from existing clinical trials to determine safety and efficacy of MTX in the treatment of acute and chronic GVHD.

## 2. Materials and Methods

### 2.1. Data Sources and Endpoints

We searched the MEDLINE database from January 1985 to April 2014 using the following keywords: “graft-versus-host disease treatment” and “methotrexate,” “steroid refractory acute GVHD,” and “steroid refractory chronic GVHD.” In addition, reference lists from review articles and selected papers were hand searched. Only peer-reviewed original articles written in English language, reporting at least five or more cases of either chronic or aGVHD treated with MTX with a description of detailed diagnostic and response criteria as well as clinical endpoints, were included.

The objective of this study was to review all the available published clinical trials on the efficacy and safety of low dose MTX for management of acute and chronic GVHD.

### 2.2. Selection of the Trials

The electronic search yielded 10 published clinical trials. Two trials were excluded (one in Chinese language and one on a canine model). Eight studies were included (4 single arm prospective phase I/II studies and 4 retrospective studies). The studies included 238 patients, and the related clinical data are listed in Table 1. Data from 17 patients were shared between two publications by the same group, and data from the more recent study were used.

### 2.3. Data Extraction and Quality Assessment

Two reviewers using a standardized approach independently conducted data extraction. Data for authors names, journal, year of publication, sample size, MTX dose and duration of treatment, median age of patients, sex, GVHD subtype, additional immunosuppressive drugs used, response to MTX therapy in different GVHD subtypes, numbers of patients assessable for 1 year overall and disease-free survival, and information pertaining to study design were collected. Evaluation of response to MTX treatment in steroid refractory or naïve acute and chronic GVHD was the primary endpoint, whereas MTX toxicity in these patients was the secondary endpoint.

### 2.4. Patients and Transplant Procedure

The studies reported a consecutive series of 238 adult and pediatric patients with both acute and chronic GVHDs after allo-SCT between 1995 and 2013, and a summary of demographic data is provided in Table 2.

### 2.5. Diagnosis and Grading of GVHD

The diagnosis and grading of both aGVHD and cGVHD were based on established clinical criteria and were uniform among all studies. Diagnosis of aGVHD was reported for 113 patients; 59 patients were refractory to steroid therapy, whereas the remaining 54 were steroid naïve at the time of MTX therapy. Although most of the patients had low-grade aGVHD, comprised between grades I and II (*n* = 67), a more severe aGVHD, classified as grade II or IV, was present in 46 patients. The skin, gastrointestinal tract (GIT), and liver were involved in 89, 57, and 21 patients, respectively. Diagnosis of cGVHD was reported for 125 patients; 39 patients were refractory to steroid therapy, whereas 86 patients were steroid naïve. Extensive cGVHD was present in 85 patients, and 40 patients exhibited limited cGVHD symptoms. Organs involved were the skin (*n* = 78), oral mucosa (*n* = 40), GIT (*n* = 8), liver (*n* = 69), and lungs (*n* = 2).

### 2.6. Methotrexate Dose and Schedule

All studies with the exception of those carried out at the University of Beijing were conducted with weekly administration of MTX, either orally or intravenously. The median dosage was 7.5 mg/m^2^/week (range 3–15 mg) for a median of 4 doses (range 3–50) for treatment of cGVHD. Identical dosage for a median of 3 doses (range: 2–60) was used in aGVHD treatment. At the University of Beijing, two initial doses of MTX were administered in the first week, followed by weekly doses administered until response; a patient was defined as nonresponsive after unsuccessful administration of 6 doses. In all studies, MTX was administered in combination with steroids or following treatment with other immunosuppressive drugs.

### 2.7. Evaluation of Response and Toxicity

A complete response (CR) was defined as complete disappearance of all clinical manifestations of GVHD. For aGVHD, partial response was defined as incomplete disappearance of symptoms, with a grade decrease of a minimum of one stage in at least one target organ. For cGVHD, a partial response (PR) was defined as a change from extensive to limited stage or a higher than 50% improvement in objective parameters of cGVHD manifestations, such as surface area involvement of skin and transaminase or bilirubin level. A response in oral cGVHD required symptomatic improvement and physician assessment. Overall response (OR) included CR and PR. Patients were considered to have no response (NR) or treatment failure, if GVHD progressed or failed to improve (stable disease) or disease progressed on treatment. The common terminology criteria (CTC) for adverse events, version 3.0, were used to grade the severity of side effects [42]. 

The results and discussions of each study were reviewed for the use/tapering off of other immunosuppressive agents after start of MTX therapy whenever available.

## 3. Results

### 3.1. Response

An OR to MTX treatment in aGVHD was reported in 79 out of 113 patients (69.9%). CR was observed in 67 out of 113 patients (59.2%) while PR was observed in 35/113 patients (10.6%). The response was better in patients with lower grade aGVHD (grades I-II) as compared to patients with grades III-IV (OR 50/58 (86%) and 11/20 (55%), resp.). The response was variable at different sites of aGVHD involvement. Response was observed at a higher frequency in skin involvement where OR was observed in 72/89 patients (80.6%), whereas OR was observed in 35/57 and in 11/23 patients with GIT or liver involvement, respectively (Table 3).

The OR of cGVHD to MTX treatment was reported in 97 out of 125 patients (77.6%); 62/125 patients showed CR (49.6%), whereas 35/125 patients (28%) showed PR. The response was better in 33/40 (82.5%) patients with limited than in 62/85 (72.9%) patients with extensive cGVHD. The response was also variable at different sites of cGVHD involvement, and the best response was observed in cutaneous (60/78 patients (77%)) than in mucosal lesions (17/40 (42.5%)), GIT involvement (4/8 (50%)), and hepatic involvement (50/69 (72%)).

The response in patients with single organ involvement was better than in patients with multiple organ involvement. An OR was observed in 47/60 (78.3%) and 61/73 (83.5%), and among these patients CR was observed in 47/60 (78.3%) and 49/73 (67.1%) of patients with single organ involvement aGVHD and cGVHD, respectively. aGVHD patients with multiorgan involvement exhibited OR of 24/38 (63.1%), and among these patients, those with CR were 19/38 (50%). Patients with cGVHD and multiorgan involvement presented an OR of 32/41 (78%), and CR was observed in 13/41 (31.7%) patients.

### 3.2. Toxicity

Overall, the low dose of MTX used in these studies was well tolerated with low incidence of grades III-IV toxicity. Hematologic toxicity of grades III-IV was observed in 47/113 aGVHD patients (41.5%) and in 22/125 cGVHD patients (17.6%). In one study, grade III elevation of transaminases was observed in a small number of patients with aGVHD (4/113 (3.5%)). Although no grades III-IV toxicity was observed for other organs, the hematologic, hepatic, mucosal, and pulmonary tissues presented frequently with grades I-II toxicity.

Mild impairment of renal functions was reported in only 6 patients whom remained stable without further intervention.

Folinic acid rescue after MTX was not reported in any of the reviewed trials.

### 3.3. Survival

Although median survival varied among studies, we attempted to calculate survival at 1 year following MTX treatment for both forms of GVHD. The OR of aGVHD and cGVHD patients after one year of MTX therapy was 84/113 (74.3%) and 115/125 (92%), respectively. No patient with cGVHD died from progression of GVHD (no response), whereas progression of aGVHD while receiving MTX therapy was the cause of death in 11/113 (10%) patients. Other causes of death were disease relapse, observed in 10/113 and 5/125 patients with aGVHD and cGVHD, respectively, and infections, observed in 7/113 and 5/125 patients with aGVHD and cGVHD, respectively. An additional 3 patients with aGVHD died because of hemorrhage, heart failure, and multiorgan failure.

## 4. Discussion

GVHD is a major cause of posttransplant morbidity and mortality. Steroid based immunosuppressive regimens are the gold standard treatment for GVHD [23]. Steroid refractory GVHD is a critical medical condition that carries higher risks of morbidity and mortality than common GVHD. To date, there is no sufficient evidence to guide the choice of a second-line treatment for steroid refractory GVHD [22]. Selection of a second-line treatment regimen should take into consideration the overall health of the patient, the previous immune suppression regimen, and the expected toxicity of the chosen regimen.

MTX is a safe and effective prophylactic treatment for early prevention of GVHD after allo-SCT [7]. Recent studies suggested that low doses of MTX exhibit antimitotic effects and can induce a sustained suppression of T-cell activation, supporting use of MTX as a GVHD therapy [33]. The safety of MTX for GVHD treatment was confirmed in this report, since most of the toxicities observed in the literature were of grade I or II. Hematologic cytopenia was the most common complication, whereas mucosal, GIT, hepatic, and pulmonary involvement was reported sporadically. Cytopenia of grades III to IV was reported less frequently than its milder forms and occurred mainly in aGVHD, possibly because of the vulnerability of the newly engrafted hematopoietic tissue. In cGVHD, no grades III-IV hematologic toxicity was observed, and the most reported complication was the suppression of blood counts. In agreement with other studies reporting MTX use for GVHD prophylaxis hepatic grades III-IV toxicity was reported in only 3.5% of aGVHD patients, reflecting the relative safety of MTX in the context of aGVHD [43, 44].

Although immune reconstitution was not assessed uniformly, the relatively low incidence of fatal infectious complications, especially in the context of severe immune suppression dictated by GVHD and its treatment, denotes that MTX did not impair immune reconstitution in those patients. O'Meara et al. reported that maintenance therapy of childhood acute lymphoblastic leukemia with MTX can cause a profound and rapid short-term drop in T-cell subsets, which reverted to pre-MTX levels one week after administration [45]. The reported T-cell suppression might be responsible for the rapid response of GVHD to MTX, which is followed by early immune reconstitution and is characterized by fewer fatal complications due to infections.

MTX was effective in treatment of both aGVHD and cGVHD with overall response rates of 69.9% and 77.6%, respectively. Better responses were observed in certain groups of patients, including those with lower grade GVHD, cutaneous involvement, and isolated organ involvement. In the multivariate analysis conducted by Huang et al. on 86 cGVHD patients treated with MTX, the only significant factor related to higher CR rate was single organ involvement [39]. The better response in these patients might be related to less severe tissue damage and the higher regenerative potential of skin in comparison to other organs, such as liver and lungs. However, this conclusion is speculative due to the lack of histopathology of affected tissues before and after treatment. CR was observed more frequently in aGVHD (59.2%) than in cGVHD (49.6%) patients, and this event may be explained by the higher proportion of the more aggressive disease in cGVHD (68%) than in aGVHD (40.7%) patients.

The high response rate to MTX treatment translated into better utilization of immunosuppressive agents, so that 69.2% and 79.2% of aGVHD and cGVHD patients, respectively, were able to either discontinue treatment with or reduce the dose of concomitant immunosuppressive agents such as corticosteroids. This is an additional advantage of MTX therapy for GVHD that is expected to reduce long-term complications and increase the quality of life of affected individuals.

The data pooled in this review suffer from several limitations. Patients were not randomized into cohorts treated with or without MTX. Patient selection criteria were not uniform, so that the studies reviewed presented high variability in factors such as initial treatment, use of different conditioning regimen, pre- and posttransplant immune suppression protocols, and graft source and HLA matching. Furthermore, there is no objective way to quantitatively assess and compare responses across the studies and the relatively small patient numbers did not allow subgroup analysis and generation of more robust data.

In conclusion, pooling of data from published studies reporting use of MTX for treatment of aGVHD and cGVHD highlights the potential of MTX as a GVHD therapy and as a steroid sparing agent. However, prospectively randomized studies with higher patient numbers are needed to confirm the value of MTX for GVHD treatment.

## Figures and Tables

**Table 1:** Studies included in the pooled analysis.

Study	Patients	Methotrexate	GVHD prophylaxis/treatment	Response	Survival	Toxicity	Reference
Giaccone et al.	Steroid refractory cGVHD *N* = 14 Progressive cGVHD *N* = 8 Unimproved cGVHD *N* = 5 Previously untreated progressive cGVHD *N* = 1	Dose = 10 (7.5–15) mg/m^2^/w Median number of doses = 25 (3–50) doses PO *N* = 12 IV *N* = 2	N/A	OR: 10/14 (71%) Reduced previous IS: *N* = 4 Continued the same IS: *N* = 5 Increased previous IS: *N* = 4 Decided to discontinue IS: *N* = 1	Median observation 25 w OS: 13/14 Cause of death: relapsing leukemia *N* = 1	Limited to grades I-II (i) Hematologic toxicity (ii) Hepatic (iii) GIT (iv) Neurological	[35]

de Lavallade et al.	*N* = 20 (i) Steroid refractory aGVHD *N* = 12 (ii) Steroid refractory cGVHD *N* = 8	Dose = 5 mg/m^2^/w IV Median number of doses = 4 doses	Prophylaxis: (i) CSA/MMF *N* = 11 (ii) CSA *N* = 9 Treatment: Methyl prednisolone *N* = 12 CSA *N* = 8	OR: aGVHD 7/12 (5 CR and 2 PR) cGVHD 6/8 No loss of response to MTX *N* = 12	Median observation 272 (22–558) days Early death from aGVHD *N* = 5 Disease progression *N* = 10	Grade ≥2 cytopenia *N* = 14	[37]

Vettenranta et al.	*N* = 5 aGVHD of skin stages I-II	Dose = 7–15 mg/m^2^/w Number of doses = 2 doses	CSA/MTX	OR: 5/5	Mean observation 10 (7–17.5) months No mortality	G II cytopenia *N* = 1/5	[34]

Huang et al.	*N* = 38 (i) aGVHD *N* = 15 grade II (ii) cGVHD *N* = 17 (skin *N* = 13, liver *N* = 14, and thrombocytopenia *N* = 12) (i) acute/chronic GVHD *N* = 4 (ii) post-DLI *N* = 2	Dose = 5–10 mg/m^2^/3-4 days till response, treatment failure, toxicity, or intolerance Total dose of MTX: (i) aGVHD 25 mg (5–60) (ii) cGVHD 40 mg (15–70) (iii) a/c GVHD 55 mg (45–65)	CSA/MMF/MTX *N* = 36 CSA/methyl prednisolone *N* = 2	Acute and acute/chronic GVHD OR: 18/19 (94.7%) Chronic and acute/chronic GVHD OR: 16/21 (76.2%) Limited cGVHD OR: 5/9 (55.6%) Extensive cGVHD OR: 11/12 (91.7%) Quiescent onset cGVHD OR: 10/11 (90.9%) De novo presentation cGVHD OR: 6/10 (60%) Post-DLI cGVHD OR: 2/2 (100%) Response by organ: (i) Skin OR 100% (ii) Gut 75% (iii) Mouth 75% (iv) Eye 100% (v) Liver 55.6%	Median follow-up 24 (8–38) months OS: 35/38 Causes of death: multiple organ failure, pneumonia, and relapse	Hematologic toxicity > grade II (i) aGVHD group *N* = 3/19 (15.8%) (ii) cGVHD group *N* = 3/21 (14.3%)	[36]

Inagaki et al.	*N* = 27 (i) Steroid refractory/dependent aGVHD *N* = 10 (ii) Steroid refractory/dependent cGVHD *N* = 17	Dose = 3–10 mg/m^2^/w (i) Median number of doses for aGVHD = 5 (1–7) doses (ii) Median duration for cGVHD = 18 months (1–68)	FK506/MTX *N* = 17 CSA/MTX *N* = 3 CSA *N* = 7	OR: aGVHD 7/10 cGVHD 10/17 (CR *N* = 4, PR *N* = 6) Response by organ: (i) Skin OR 7/8 (88%) (ii) Gut 3/6 (50%) (iii) Liver 1/4 (25%) (iv) Eye 100% (v) cGVHD oral mucosa 8/14 (57%) (vi) cGVHD of liver 6/11 (55%) (vii) cGVHD of skin 5/12 (42%)	Median observation 18 (0–68) months	Grades III and IV hematologic or hepatic toxicity *N* = 6/27	[38]

Wang et al. 1	*N* = 86 (i) Limited cGVHD *N* = 38 (ii) Extensive cGVHD *N* = 48	Dose = 15 mg PO or 10 mg IV Q 3-4 days for 2 doses then Q weekly till response, treatment failure, toxicity, or intolerance Minimum of 3 doses were given to all patients	CSA *N* = 38 CSA/prednisone *N* = 25 Prednisone *N* = 2 CSA/prednisone/MMF *N* = 5 None *N* = 6	OR: 71/86 (83%) CR: 53/86 (62%) Flare-up after initial response 25/71 (35%) Response to retreatment with MTX 17/25		(i) Cytopenia = grade II *N* = 5 (ii) Oral mucosal toxicity *N* = 7/86 (iii) No grade IV toxicity	[39]

Wang et al. 2	*N* = 32 First line therapy of aGVHD	Dose = 15 mg PO or 10 mg IV Q 3-4 days for 2 doses then Q weekly till response, treatment failure, toxicity, or intolerance	Methyl prednisone 0.5 mg/kg/day	OR: 26/32 (81%) aGVHD of skin 23/26 (88%) aGVHD of the liver 3/4 (75%) aGVHD of the gut 9/11 (81%)		Cytopenia grade III *N* = 3	[40]

Inagaki et al. 2	*N* = 35 Steroid refractory aGVHD	10 mg/m^2^ IV Q weekly Median number of doses *N* = 5 (1–48) doses	CSA/MTX *N* = 4 FK506/MTX *N* = 31	OR: *N* = 16/35 CR: *N* = 13/35 PR: *N* = 3/35 aGVHD of skin *N* = 16/23 aGVHD of liver *N* = 3/4 aGVHD of GIT *N* = 11/23	Median follow-up: 60 (7–124) months OS: 22/35 OS at 6 months: 82.9% OS at 12 months: 65%	Cytopenia > grade II *N* = 26/35 Transaminases > grade II *N* = 2/35 Infectious complications *N* = 17/35	[41]

aGVHD: acute graft-versus-host disease; cGVHD: chronic graft-versus-host disease; MTX: methotrexate; N/A: not assessable; CSA: cyclosporin A; MMF: mycophenolate mofetil; OR: overall response; CR: complete response; PR: partial response; NR: no response; IS: immune suppression; OS: overall survival.

**Table 2:** Patient characteristics.

	Giaccone et al., 2005 [35]	de Lavallade et al., 2006 [37]	Vettenranta et al., 1997 [34]	Huang et al., 2005 [36]	Wang et al., 2009 [39]	Wang et al., 2011 [40]	Inagaki et al., 2008 [38]	Inagaki et al., 2014 [41]
Number	*N* = 14	*N* = 20	*N* = 5	*N* = 42	*N* = 86	*N* = 32	*N* = 27	*N* = 35

Age in years	44 (27–66)	51 (45–60)	5.5 (3.6–11.6)	GIT32.5 (13–45)	30 (8–50)	28 (3–46)	9 (2–16)	7 (0–18)

Gender (M/F)	8/6	NA	2/3	29/13	53/33	21/13	12/15	

Diagnosis HM Solid tumor BH	*N* = 14	*N* = 17 *N* = 3	*N* = 5	*N* = 42	*N* = 86	*N* = 32	*N* = 23 *N* = 4	*N* = 31 *N* = 4

Conditioning Myeloablative Nonmyeloablative	*N* = 12 *N* = 2	*N* = 14 *N* = 6	*N* = 5	*N* = 42	*N* = 86	*N* = 32	*N* = 26 *N* = 1	*N* = 33 *N* = 2

HLA matching Less than 1 locus mismatch More than 1 locus mismatch	*N* = 12 *N* = 2	N/A N/A	*N* = 5	*N* = 30 *N* = 12	*N* = 40 *N* = 46	*N* = 9 *N* = 23	*N* = 25 *N* = 2	*N* = 21 *N* = 14

GVHD prophylaxis CSA/MTX CSA/MMF CSA CSA/MMF/MTX CSA/MMF/MPO CSA/MPO FK506/MTX MPO None								
	*N* = 14		*N* = 5					*N* = 4
		*N* = 11			*N* = 5			
		*N* = 9			*N* = 38			
		GVHD prophylaxis		*N* = 40		*N* = 32		
				*N* = 2	*N* = 10			
					*N* = 25			
					*N* = 2			*N* = 31
					*N* = 6			

Type of GVHD	Steroid refractory cGVHD (14)	Steroid refractory aGVHD (12) Steroid refractory cGVHD (8)	Acute cutaneous GVHD steroid naïve (5)	Steroid refractory aGVHD (19) Steroid refractory cGVHD (21)	Steroid refractory cGVHD	Steroid naïve cGVHD	Steroid refractory aGVHD (10) Steroid refractory cGVHD (17)	Steroid refractory aGVHD

GVHD by organ involved Skin Mucous membrane GIT Cytopenia Eyes Liver Lung	11 14 — 2 1 — 2	20 6 10 — 5 13 1	5 — — — — — —	32 — 15 12 — 14 —	50 12 2 — 7 52 —	26 — 11 — — 4 —	12 14 7 — 2 15 —	23 — 23 — — 4 —

HM: hematologic malignancy; BH: benign hematologic diseases; HLA: human leucocyte antigen; MPO: methyl prednisolone; GIT: gastrointestinal tract; aGVHD: acute graft-versus-host disease; cGVHD: chronic graft-versus-host disease; MTX: methotrexate; N/A: not assessable; CSA: cyclosporin A; MMF: mycophenolate mofetil.

**Table 3:** Response and outcome data.

	cGVHD	aGVHD
Number	*N* = 125	*N* = 113

Age	30 (2–60) years	35 (2–59)

Type Steroid refractory Steroid naïve	*N* = 39 *N* = 86	*N* = 59 *N* = 54

Stage	Limited *N* = 40 Extensive *N* = 85	I-II *N* = 67 III-IV *N* = 46

Previous immune suppression MTX containing Non-MRTX containing None	*N* = 34 *N* = 105 6	*N* = 113 *N* = 51 0

MTX Dose Number of doses	7.5 (3–15) mg /m^2^/w 4 (3–50)	7.5 (3–15) mg/m^2^/w 3 (2–60)

Response OR CR PR NR	97/125 (77.6%) 62/125 (49.6%) 35/125 (28%) 28/125 (22.4%)	79/113 (69.9%) 67/113 (59.2%) 12/113 (10.6%) 34/113 (30%)

Response by stage	Limited 33/40 (82.5%) Extensive 62/85 (72.9%)	I-II 50/58 (86%) III-IV 11/20 (55%)

**Response by organ** **Skin** **Oral mucosa** **GIT** **Liver** **Pulmonary**	60/78 (77%) 17/40 (42.5%) 4/8 (50%) 50/69 (72%) 2/2 (100%)	72/89 (80.8%) 35/57 (61.4%) 11/23 (47.8%)

**Response by number of organs involved** **Single organ** **OR** **CR** **Multiorgan involvement** **OR** **CR**	61/73 (83.5%) 49/73 (67.1%) 32/41 (78%) 13/41 (31.7%)	47/60 (78.3%) 47/60 (78.3%) 24/38 (63.1%) 19/38 (50%)

**Discontinue or reduce IS** **Continue or increase other IS**	99/125 (79.2%) 26/125 (20.8%)	67/113 (59.3) 46/113 (40.7%)

**Toxicity ** **Hematologic toxicity ** **Hepatic toxicity** **Mucosal toxicity ** **Pulmonary toxicity**	Grade 3 or more *N* = 22/125 (17.6%) <Grade 2 <Grade 2 <Grade 2	Grade 3 or more *N* = 47/113 (41.5%) Grade 3 or more *N* = 4/113 (3.5%)

**Survival at least one year after GVHD**	115/125 (92%)	84/113 (74.3%)

**Cause of death** **Uncontrolled GVHD** **Relapse** **Infectious complications** **Hemorrhage** **Multiorgan failure**	— 5/125 (4%) 5/125 (4%) — —	*N* = 11/113 *N* = 10/113 *N* = 7/113 *N* = 2/113 *N* = 1/113

HM: hematologic malignancy; BH: benign hematologic diseases; HLA: human leucocyte antigen; MPO: methyl prednisolone; GIT: gastrointestinal tract; aGVHD: acute graft-versus-host disease; cGVHD: chronic graft-versus-host disease; MTX: methotrexate; N/A: not assessable; CSA: cyclosporin A; MMF: mycophenolate mofetil; OR: overall response; CR: complete response; PR: partial response; NR: no response; IS: immune suppression; OS: overall survival.
